# Modern Therapeutics

**Published:** 1877-05-20

**Authors:** 


### BOOK NOTICES.

Modern Therapeutics.—A compendium of recent formulæ, approved treatment and specific methods in Medicine and Surgery, with an appendix on hypodermic medication inhalation, aeration, and other remedial agents and therapeutic methods of recent introduction. By George H. Napheys, A. M., M. D. Fourth edition, re-written and enlarged. Philadelphia, D. G. Brinton.

Medical men will be glad to learn that a new and revised edition of this valuable work is in hand for distribution. We are acquainted with no single production which can so nearly place in the hands of the practitioner, as a book of reference, the accumulated improvements of modern therapeutics. A new division on surgical therapeutics is added, and the section on diseases of women and children are much enlarged. Numerous valuable formulæ are embodied in the work. The diseases are alphabetically arranged under the general nosological division to which they belong. The treatment as recommended by different practitioners is stated, and that by the various hospitals in each disease, followed by a resume of the more important remedies used. We hesitate not to recommend this work as a very valuable production.

